# Synergistic heterojunction effects in Ag_3_PO_4_/SnO_2_ nanocomposites: a photocatalytic study on isoproturon degradation

**DOI:** 10.3389/fbioe.2025.1458965

**Published:** 2025-04-04

**Authors:** Rishi Ram, Bhawna, Sanjeev Kumar, Akanksha Gupta, Ravinder Kumar, Kashyap Kumar Dubey, Vinod Kumar

**Affiliations:** ^1^ School of Physical Sciences, Jawaharlal Nehru University, New Delhi, India; ^2^ Department of Chemistry, SRM Institute of Science and Technology, Delhi-NCR Campus, Ghaziabad, India; ^3^ Department of Chemistry, University of Delhi, Delhi, India; ^4^ Department of Science and Technology, Technology Bhavan, New Delhi, India; ^5^ Department of Chemistry, Gurukula Kangri (Deemed to be University), Haridwar, Uttarakhand, India; ^6^ School of Biotechnology, Jawaharlal Nehru University, Delhi, India; ^7^ Sustainable Energy and Environmental Nanotechnology Group, Special Centre for Nano Science, Jawaharlal Nehru University, Delhi, India

**Keywords:** photocatalysis, nanoparticles, photochemistry, heterojunction, pesticide

## Abstract

**Introduction:**

Pesticides such as isoproturon are widely employed and represent a considerable environmental concern. The development of sustainable and efficient degrading techniques is crucial. Photocatalytic degradation employing semiconductor materials is a compelling solution. This study examines the synergistic advantages of heterojunction formation by synthesizing, characterizing, and improving the photocatalytic efficacy of Ag_3_PO_4_/SnO_2_ nanocomposites for the degradation of isoproturon.

**Methods:**

The Ag_3_PO_4_/SnO_2_ nanocomposite was characterised using powder X-ray diffraction (PXRD), Fourier Transform Infrared Spectroscopy (FTIR), Scanning Electron Microscopy (SEM), Ultraviolet-Diffuse Reflectance Spectroscopy (UV-DRS) and X-ray Photoelectron Spectroscopy (XPS). The effective synthesis of the Ag_3_PO_4_/SnO_2_ heterojunction was confirmed by characterization data from various techniques (PXRD, FTIR, SEM, UV-DRS, XPS).

**Results and Discussion:**

Elemental mapping confirmed uniform distribution of O, P, Ag, and Sn. High-resolution mass spectrometry (HRMS) was employed to analyse degradation products. The Ag_3_PO_4_/SnO_2_ nanocomposite exhibited improved photocatalytic degradation of isoproturon compared to its precursors. In contrast to 25% for pure SnO_2_ and 41% for Ag_3_PO_4_, over 97% degradation was achieved using Ag_3_PO_4_/SnO_2_ nanocomposite within 120 min of light irradiation under identical conditions. The synergistic effects of heterojunction formation significantly enhanced isoproturon degradation using the Ag_3_PO_4_/SnO_2_ nanocomposite. The heterojunction reduces electron-hole recombination rate and enhances photogenerated charge carriers for degradation via effective charge separation. The improved photocatalytic activity is ascribed to the increased surface area of the nanocomposite. The analysis of HRMS data revealed the degradation products. The findings demonstrate the efficacy of Ag_3_PO_4_/SnO_2_ nanocomposites as photocatalysts for environmental remediation, namely in the breakdown of pesticides.

## Graphical Abstract

**Figure:**
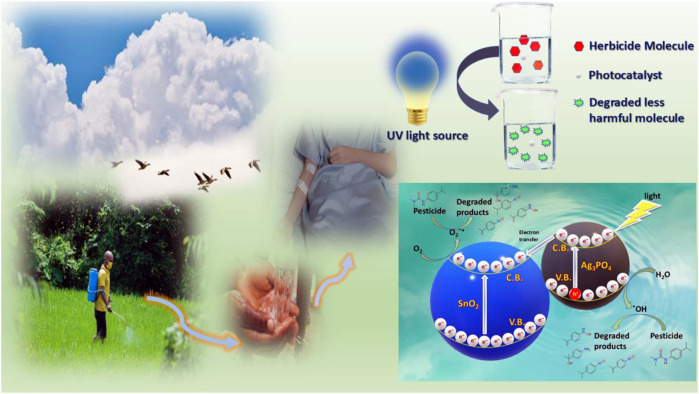

## 1 Introduction

Pesticides serve as chemical agents designed to safeguard crops from detrimental pests and diseases affecting humans. The positive ramifications of employing pesticides render them a crucial tool for upholding and elevating the global population’s quality of life. The annual consumption of pesticides across the world is about two million tons (De et al., 2014). The term pesticide conventionally includes herbicides, insecticides, rodenticides, and fungicides, etc., depending upon the targeted species (Sharma et al., 2019). Herbicides and insecticides represent the predominant types of pesticides, constituting 47.5% and 29.5%, respectively, of the overall pesticide utilization (De et al., 2014). Globally, countries like India, China, Japan, Canada, and Brazil, etc., are the major consumers of pesticides (Sharma et al., 2019). Also, most of the pesticides undergo biological magnification which means their concentration increases on moving to higher trophic level. This makes the aquatic organism hazardous for consumption. Significantly, prolonged exposure to pesticides through water ingestion can emulate the hormonal functions within the human body, thereby compromising immune response, disrupting hormone equilibrium, eliciting reproductive-related complications, imparting carcinogenic effects, and diminishing cognitive abilities, particularly among children in the developmental stage (Yadav et al., 2015).

Use of pesticides in agriculture is concerning as these chemicals are water soluble, and the rate of chemical and biological degradation is very slow. Ground and surface water are contaminated due to poor agricultural practices, reckless dumping of empty containers, and equipment washing (Spliid and Køppen, 1998). In the interest of public health protection, various nations have published guidelines for permissible levels of pesticides in drinking water. Discrepancies in permissible pesticide levels in drinking water may arise due to socio-economic, dietary, geographical, and industrial variations (Hamilton et al., 2003). Agricultural soils around the world are heavily treated with phenyl urea herbicides and Isoproturon (IPU) to control weeds. IPU is a systemic herbicide used to manage broad-leaved weeds and annual grasses in agricultural fields. As a result, various methods for removing these contaminants from water have been developed such as ozonation (Magara et al., 1995), electrical discharge (Malik et al., 2001), activated carbon nanofiltration (Appleman et al., 2013), etc. The application, efficiency, and cost of these operations are all constrained by their very nature (Yeoh et al., 2022). Ozonation does not lead to mineralisation of organic contaminants (Von Sonntag and Von Gunten, 2012) and oxidised byproducts are formed (von Gunten, 2003) while electrical discharge has shown problems in scaling up (Zeghioud et al., 2020). One of the most promising methods to treat contaminated water is photocatalysis. Photocatalysis is an environmentally friendly degradation method which utilizes a photocatalyst to decompose pollutants using oxidation or reduction process in the presence of light irradiation (Ahmed et al., 2021; El-Saeid et al., 2021; Dhenadhayalan et al., 2022). AOPs typically rely on the production of OH^•^ radicals that interact with organic contaminants to cause gradual deterioration and ultimately whole-body mineralization (Verma et al., 2014). An efficient photocatalyst must have various characteristics, including non-toxicity, high efficiency, low cost, recyclable, and an effective light absorber. Metal oxide nanoparticles have semiconducting characteristics and having large surface area. As a result, these materials serve an important role as photocatalysts for the degradation of contaminants.

There have been a lot of photocatalysts used for photocatalysis over the years. But recently a p-type semiconducting material silver phosphate (Ag_3_PO_4_), has witnessed a surge in popularity due to its capability of utilizing visible light to decompose water molecules and disintegrate organic pollutants (Kamiuchi et al., 2010). Nevertheless, the practical application of Ag_3_PO_4_ is hindered by photo corrosion due to reduction of Ag^+^ ions by the photoexcited electrons during the photocatalytic process (Chen et al., 2015). A widely employed technique to mitigate photo corrosion and enhance the overall photocatalytic efficiency involves the integration of Ag_3_PO_4_ with other semiconductors, resulting in the formation of an Ag_3_PO_4_-semiconductor composite. Zheng et al. (2017) reported higher efficiency of Ag_3_PO_4_/Bi_4_Ti_3_O_12_ as compared to pure Ag_3_PO_4_ and Bi_4_Ti_3_O_12_ for breakdown of Rhodamine B (RhB) under simulated solar irradiation. The degradation rate of composite containing 10% molar Bi_4_Ti_3_O_12_ was 2.6 times higher as compared to pure Ag_3_PO_4_. Formation of heterojunction facilitate increased charge separation between both the molecules which resulted in high photocatalytic efficiency. Qi et al. studied the photocatalytic performance of Ag_3_PO_4_-BiOCl_1-x_Br_x_ for the degradation of phenol under artificial sunlight (Qi et al., 2018). Ag_3_PO_4_ has recently been found to couple with additional wide-band gap semiconductors, including WO_3_ (Lu et al., 2017), TiO_2_ (Liu and Perng, 2020), and ZnO (Yu et al., 2020). In order to create effective photocatalysts, much effort has been put into synthesising Ag_3_PO_4_-semiconductor composites with acceptable band gap. One of the most significant n-type semiconductors is tin dioxide (SnO_2_). By interacting with other semiconductors, SnO_2_ is known to be effective owing to formation of p-n junctions (We et al., 2019; Wen et al., 2017). Additionally, there aren't a lot of studies on the synthesis of Ag_3_PO_4_/ SnO_2_ composites and the analysis of their characteristics in the literature (Liu et al., 2020; Zhang et al., 2012; Li et al., 2019a). For instance, when subjected to visible light irradiation, the Ag_3_PO_4_/SnO_2_ composite synthesised by Zhang et al. (Liu et al., 2020) showed excellent photocatalytic activity to facilitate the photodegradation of methyl orange dye. The efficient e^−^-h^+^ separation was attributed for the enhancement in the photocatalytic performance. Li et al. also synthesized Ag_3_PO_4_/SnO_2_ catalyst using hydrothermal method (Li et al., 2019a). The catalyst exhibits significantly improved tetracycline degradation under visible light irradiation compared to Ag_3_PO_4_ and SnO_2_. Optimum conditions yielded a 74% degradation within 60 min. Gabriela et al. also synthesized Ag_3_PO_4_/SnO_2_ composite with varying SnO_2_ ratios (Silva et al., 2021). Min Liu et al. prepared Ag_3_PO_4_/SnO_2_ heterojunctions on carbon cloth using a simple two-step process (Liu et al., 2020). The synthesis method involved deposition of SnO_2_ on carbon cloth followed by *in-situ* growth of Ag_3_PO_4_ nanoparticles. The catalyst demonstrated significantly improved photocatalytic activity degrading 95% of RhB within 60 min.

In this study, Ag_3_PO_4_/ SnO_2_ nanocomposite was prepared via hydrothermal method. The composite was analysed using various analytical techniques. The composite was used for the photocatalytic degradation of IPU. The nanocomposite degraded 97% of pesticide in 120 min. This work opens up new pathways for the utilisation of Ag_3_PO_4_/ SnO_2_ nanocomposite in environmental remediation methods.

## 2 Materials and methods

### 2.1 Materials

The reagents used in this study were commercially available. The chemicals were directly used without further purification. SnCl_2_•2H_2_O (≥99.0% purity) and CH_3_OH (≥99.0% purity) from Merck Chemicals, H_2_O_2_ (Fisher Scientific, 30% w/v), AgNO_3_ (99.9% purity) from Rehsiff scientific, and Na_2_HPO_4_ (99.5% purity) were purchased from Fisher scientific.

### 2.2 Synthesis of Ag_3_PO_4_/ SnO_2_ nanocomposites

For the synthesis of Ag_3_PO_4_/SnO_2_ nanocomposites, 1.32 mM methanolic solution of SnO_2_ (S.I.1) was sonicated for 60 min. To this solution, 3 mM aqueous solution of AgNO_3_ (S.I.2) was added and sonicated for 40 min. It was followed by dropwise addition of 49.3 mM aqueous Na_2_HPO_4_ solution. Subsequently, the reaction solution was kept in Teflon vessel in hydrothermal at 150°C for 15 h. Then the synthesized Ag_3_PO_4_/SnO_2_ nanocomposite were washed with water several times and dried in an oven at 80°C. Pure Ag_3_PO_4_ and SnO_2_ nanoparticles were synthesized using same method at same conditions and the synthesis method have been described in Supplementary Material.

### 2.3 Photocatalytic experiments

A specially designed photocatalytic reactor equipped with water circulatory system was used for the study of IPU degradation as described in our previous work (Kumar et al., 2022). Water circulation around the reactor helps in cooling. A 125-W mercury lamp (Philips, India) was utilised as a source of UV light (Bhawna et al., 2023). A 100 mL solution of 50 μM isoproturon was taken in photocatalytic reactor along with 100 mg of synthesized photocatalyst. The solution was stirred for 30 min in dark in order to reach the adsorption/desorption equilibrium before light irradiation. A 5 mL of the IPU solution was pipetted out at regular intervals and was centrifuged. The UV-visible spectrum was recorded to check the degradation efficiency.

## 3 Results and discussion

### 3.1 Powder X-ray diffraction analysis

**FIGURE 1:**
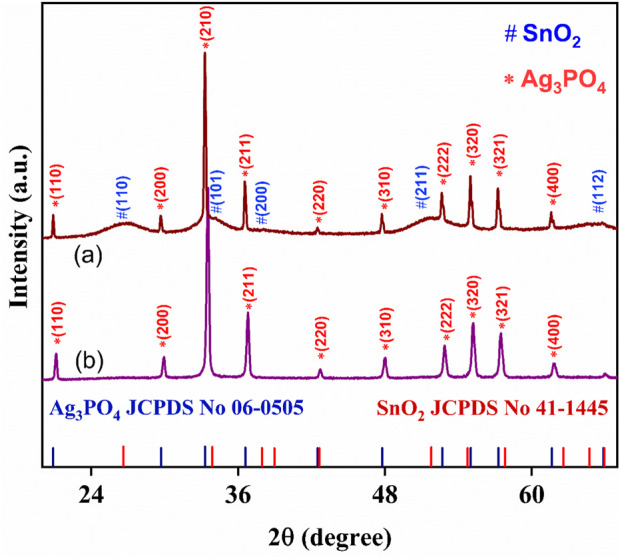
Powder XRD patterns of (a) Ag_3_PO_4_/SnO_2_ nanocomposite and (b) Ag_3_PO_4_.

The Powder X-Ray Diffraction pattern of Ag_3_PO_4_/SnO_2_ nanocomposite (NC) and is pure Ag_3_PO_4_ depicted in Figures 1a, b, respectively. The characteristic planes (110), (101) and (211) corresponding to SnO_2_ phase reveal a tetragonal crystal system with a rutile structure having space group *P4*_*2*_ */mnm* (JCPDS No. 41-1445) (Kumar et al., 2019). Similarly, the identified feature planes of Ag_3_PO_4_, (210) and (211) exhibits a well-defined crystalline body-centred cubic structure with the space group $P4\bar{3}n$ (JCPDS No. 06-0505) (Li et al., 2019a). (28). Additionally, the PXRD peaks of Ag_3_PO_4_/SnO_2_ nanocomposite corresponding to Ag_3_PO_4_, exhibit a shift towards a lower diffraction angle. This shift is attributed to the presence of strain within the lattice structure, resulting due to alterations in interplanar distances. Furthermore, a slight change is observed in peak intensities between the Ag_3_PO_4_/SnO_2_ nanocomposite and pure Ag_3_PO_4_ nanoparticles. Variation in peak intensity is directly linked to the level of crystallinity (Sen et al., 2020), indicating that the Ag_3_PO_4_ phase in the nanocomposite possesses a slightly lower degree of crystallinity. This also indicates that lattice distortion of Ag_3_PO_4_ and SnO_2_ occurs during synthesis due to interaction between the two phases (Zhu et al., 2020). The absence of additional peaks in diffraction pattern indicates the successful formation of Ag_3_PO_4_/ SnO_2_ nanocomposite. A comparative PXRD pattern for pure SnO2, pure Ag_3_PO_4_ and Ag_3_PO_4_/ SnO_2_ nanocomposite has been provided in Supplementary Figure S1. Supplementary Figure S2 shows the W-H plot after linear fitting. From W-H plot, the average crystallite size was calculated to be 95.62 nm. Intrinsic strain (1.47 × 10^−3^) develops due to defects in crystal structure. Due to insertion of ions in crystal lattice, lattice expansion or contraction occurs during formation of nanocomposite. Due to this intrinsic strain and defects are generated.

### 3.2 Morphology study

**FIGURE 2:**
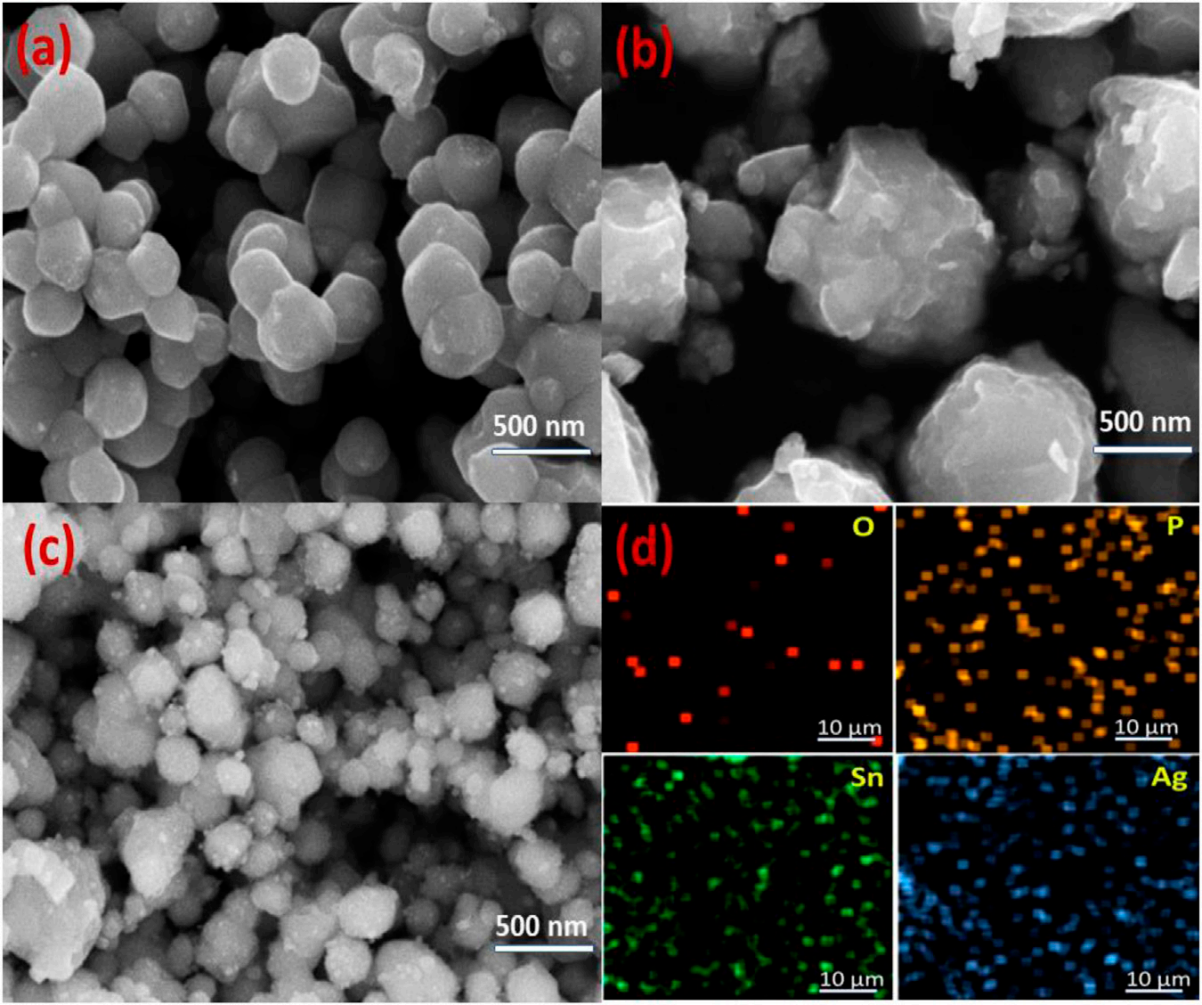
SEM images of **(a)** Ag_3_PO_4_ NPs **(b)** SnO_2_ NPs **(c)** Ag_3_PO_4_/SnO_2_ NPs and **(d)** Elemental mapping of Ag_3_PO_4_/SnO_2_ nanocomposite.

SEM imaging was used to analyse the topography of synthesised NCs (Figure 2). Figure 2A shows that the nanoparticles of Ag_3_PO_4_ are agglomerated and spherical shaped. The combined effect of different attractive-repulsive forces and some weak forces is responsible for the nanoparticle agglomeration. The formation of irregular shaped SnO_2_ NPs is illustrated in Figure 2B. In Figure 2C shows the SEM image of Ag_3_PO_4_/SnO_2_ nanocomposite featuring both spherical and irregularly shaped nanoparticles. This observation highlights the amalgamation of SnO_2_ and Ag_3_PO_4_ structures, culminating in the formation of a composite material. This may be advantageous for the facile transfer of charge carriers between Ag_3_PO_4_ and SnO_2_. The uniform distribution of Sn, Ag, P, and O elements is demonstrated through elemental mapping (Figure 2D).

**FIGURE 3:**
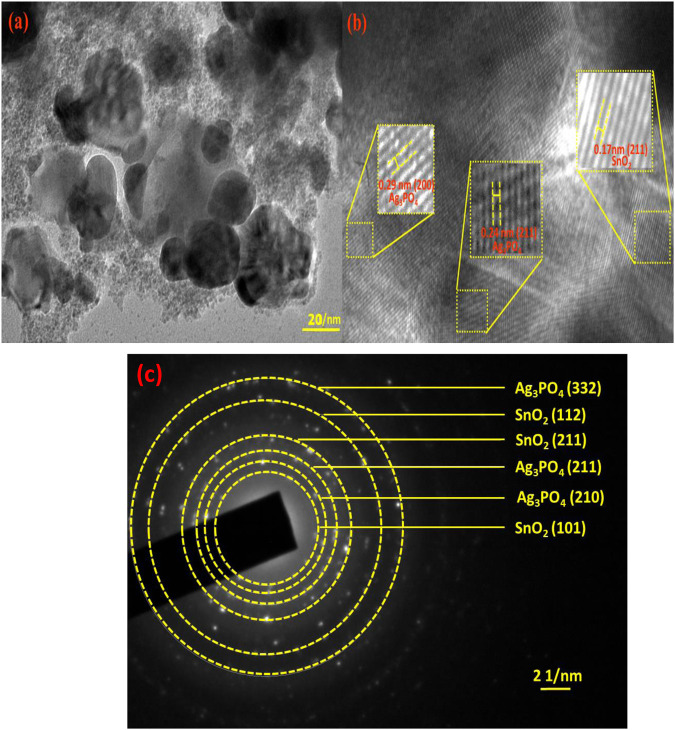
**(a)** TEM image of Ag_3_PO_4_/SnO_2_ NP_s_ **(b)** lattice fringes and **(c)** selected area electron diffraction pattern of Ag_3_PO_4_/SnO_2_ NPs.

TEM image of nanocomposite has been represented in Figure 3A. TEM image shows different types of nanoparticles ranging between 20–50 nm which are spherical as well as irregular in nature. The crystal lattice fringe spacings of 0.29, 0.24, and 0.17 nm corresponds to Ag_3_PO_4_ (200), Ag_3_PO_4_ (211), and SnO_2_ (211) planes, respectively (Figure 3B). Additionally, the selected area electron diffraction (SAED) pattern in Figure 3C, shows the highly crystalline structure of Ag_3_PO_4_ and SnO_2_.

### 3.3 XPS analysis

**FIGURE 4:**
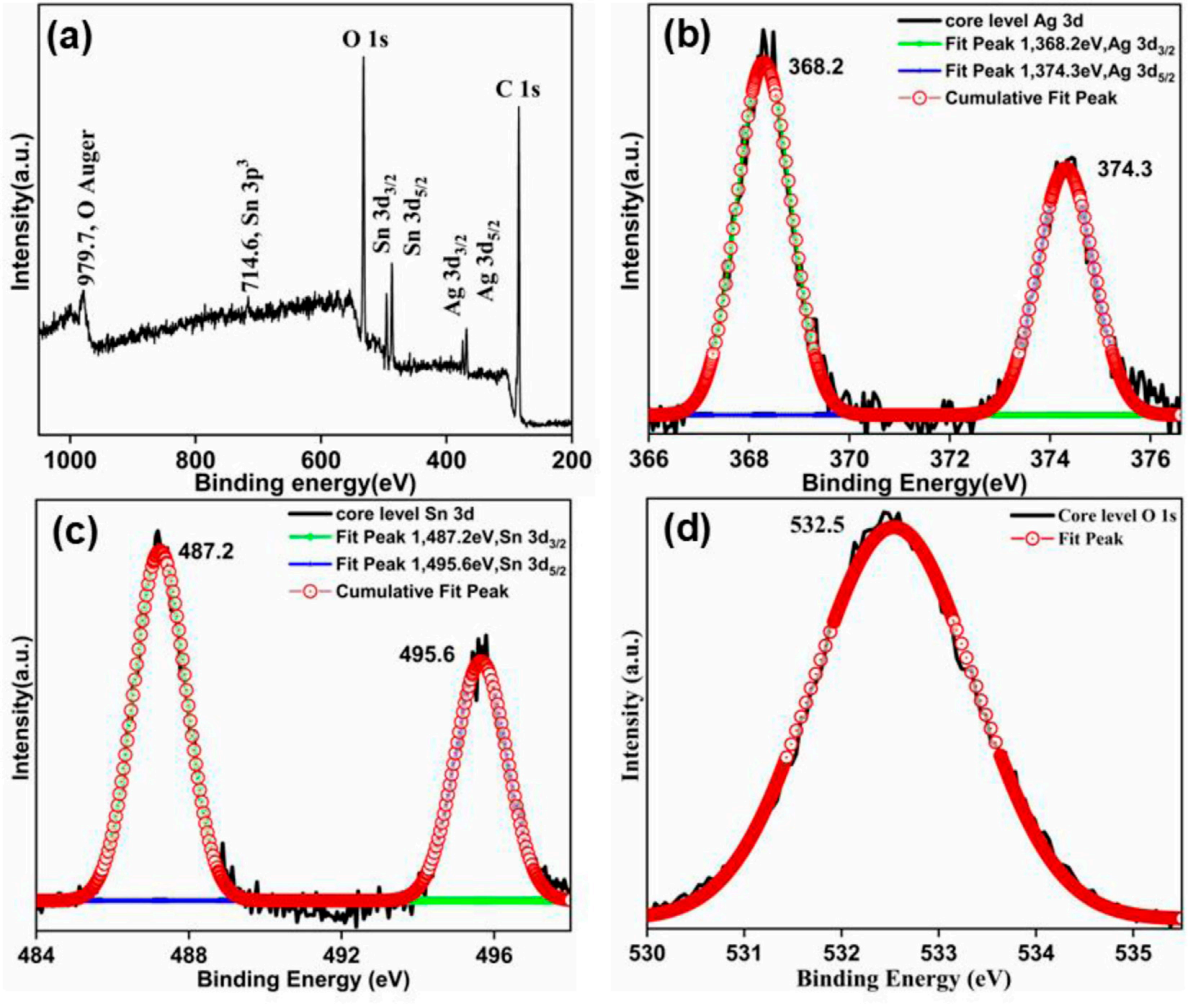
**(a)** XPS survey spectrum of Ag_3_PO_4_/SnO_2_ nanocomposite; De-convoluted XPS core level spectra of **(b)** Ag 3d **(c)** Sn 3d and **(d)** O 1s.

X-ray photoelectron spectroscopy was utilized to examine the composition and the valence state of different elements in synthesized Ag_3_PO_4_/SnO_2_ nanocomposite. The XPS analysis of the sample (Figure 4A) displayed the presence of Sn, Ag and O elements in the synthesized nanocomposite. The spectrum was calibrated with reference to the C *1s* peak.

The core level XPS spectrum of Ag *3d*, Sn *3d* and O *1s* (Figure 4) was deconvoluted using gaussian function. Ag exhibits two characterstic peaks of binding energy approximately at 368.2 eV and 374.3 eV (Figure 4B) corrosponding to Ag *3d*_3/2_ and Ag*3d*_5/2_. It confirms that Ag is present as Ag(I) (Li et al., 2019b; Chai et al., 2014; Li et al., 2019c). The XPS peak of Sn 3*d* (Figure 4C) was deconvoluted in two prominent peaks having binding energy of 487.2 eV and 495.6 eV, corresponding to the Sn *3d*_5/2_ and Sn *3d*_3/2_ states, respectively. This indicate that Sn is present as Sn^+4^ in the nanocomposite (Wang et al., 2019; Ma et al., 2019; Kamboj et al., 2022). Additionally, the deconvoluted peak of P is observed at 135 eV (Supplementary Figure S2) which is attributed to P *2p*_3/2_. The peak around 532.5 eV is attributed to O *1s* band (Figure 4D). These peaks confirms that P is present as P^5+^ (Li et al., 2019c) and O is present as O_2_- in the form of PO_4_^3-^ as well as in SnO_2_ lattice. Moreover, in the XPS spectra of Ag_3_PO_4_/SnO_2_, apart from Sn, Ag, O and P, no distinct impurity peaks were observed, which confirms the formation of pure Ag_3_PO_4_/SnO_2_ nanocomposite.

### 3.4 Brunauer-Emmett-Teller surface area determination

The Brunauer-Emmett-Teller is an important analytical method for calculating the average surface area of materials. BET characterization was performed using 80 mg Ag_3_PO_4_/SnO_2_ nanocomposite. nanoparticles.

**FIGURE 5:**
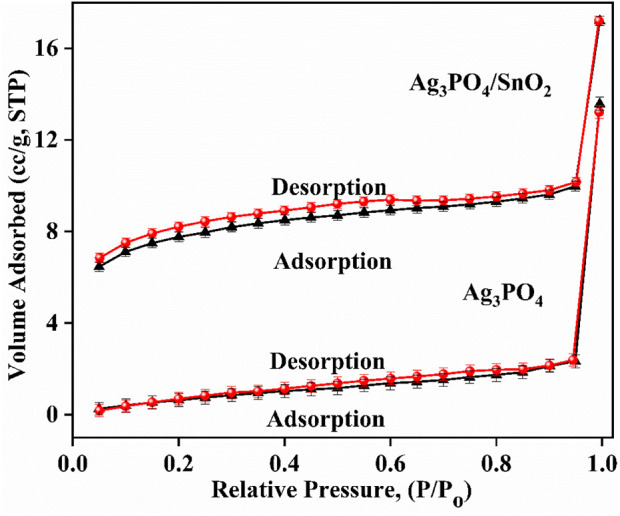
Adsorption-desorption isotherm of Ag_3_PO_4_/SnO_2_ nanocomposite and pure Ag_3_PO_4_ nanoparticles.

Figure 5 shows that the adsorption-desorption isotherms for both the Ag_3_PO_4_/SnO_2_ nanocomposite and Ag_3_PO_4_ nanoparticles are of Type IV according to IUPAC classification (Thommes et al., 2015). The hysteresis loop for Ag_3_PO_4_/SnO_2_ is slightly wider than the hysteresis loop for Ag_3_PO_4_. This suggests that the pores in the Ag_3_PO_4_/SnO_2_ material are slightly more ink-bottle shaped (Thommes et al., 2015; Yamaguchi et al., 2020; Cychosz et al., 2017) which can absorb more light and thus results in higher photocatalytic activity. Moreover, the mean pore radius, total pore volume, and average surface area of the Ag_3_PO_4_/SnO_2_ nanocomposite was found to be 1.5261 nm, 0.0266 cm^3^/g, and 37.674 m^2^/g, respectively. The mean pore radius, total pore volume, and average surface area of the pure Ag_3_PO_4_ nanoparticles are 1.706 nm, 0.021 cm^3^/g, and 7.021 m^2^/g, respectively. Table 1 summarizes the compared values from BET analysis for Ag_3_PO_4_ and Ag_3_PO_4_/SnO_2_. Table 1 depicts that the average surface area of the Ag_3_PO_4_/SnO_2_ nanocomposite is almost 5.36 times higher than that of Ag_3_PO_4_ nanoparticles (Figure 5). This increase in surface area indicates that more surface-active sites are available in Ag_3_PO_4_/SnO_2_ nanocomposite than the pure Ag_3_PO_4_.

**TABLE 1:** BET analysis of Ag_3_PO_4_/SnO_2_ nanocomposite and Ag_3_PO_4_ nanoparticles.

Sample	Average pore radius (nm)	Average surface area (m^2^g^−1^)	Total pore volume (cm^3^g^−1^)
Ag_3_PO_4_	1.706 nm	7.021	2.1 × 10^−2^
Ag_3_PO_4_/SnO_2_	1.5261 nm	37.674	2.661 × 10^−2^

### 3.5 DRS analysis

**FIGURE 6:**
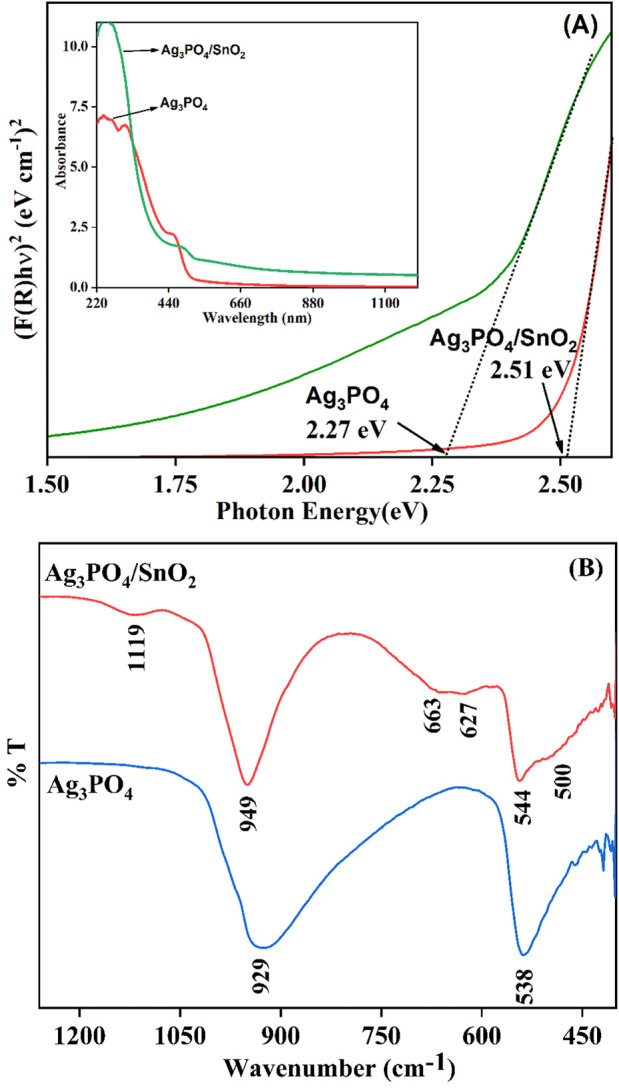
**(A)** Band gap of Ag_3_PO_4_ and Ag_3_PO_4_/SnO_2_, (inset) UV-DRS absorption spectrum of Ag_3_PO_4_ and Ag_3_PO_4_/SnO_2_ **(B)** FTIR spectrum of Ag_3_PO_4_ and Ag_3_PO_4_/SnO_2_.

Using DRS, the optical property of the Ag_3_PO_4_/SnO_2_ nanocomposite was investigated to evaluate the effect of Ag_3_PO_4_/SnO_2_ heterojunction on light absorption activity. To determine the optical band gap energy, the linear fit of the graph between (F(R)hυ)^2^ and photon energy was extrapolated using Kubelka Munk function. The observed band gaps for pure Ag_3_PO_4_, SnO_2_ and Ag_3_PO_4_/SnO_2_ composite are 2.27 eV, 3.89 eV and 2.51 eV respectively, as shown in Figure 6A. The formation of a heterojunction in between Ag_3_PO_4_ and SnO_2_ changes the overall band gap and enhance the light absorption property resulting in high photocatalytic activity.

### 3.6 FTIR analysis

FTIR spectrum was used to study the bonding characteristics of the nanocomposite (Figure 6B). The broad shoulder peak at 500 cm^−1^ corresponds to O–Sn–O stretching vibrations, and the peaks at 627 cm^−1^ is the characteristic of Sn–O bond stretching (Kumar et al., 2011). The discernible peak at 544 cm^−1^ signifies the asymmetric bending vibration associated with O=P–O bonds (Nagajyothi et al., 2019).The peak at 663 cm^−1^ indicate the P-O-P bond stretching (Mroczkowska et al., 2007). Additionally, a prominent peak observed at 949 cm^-1^ is attributed to the P=O stretching due to presence of PO_4_^3−^ ions (Nagajyothi et al., 2019). The peak at 1119 cm^-1^ represents the antisymmetric stretching of P-O bonds (Kumar et al., 2013). A pristine phase of Ag_3_PO_4_ exhibits characteristic vibrational modes at 929 cm⁻^1^ and 538 cm⁻^1^. Upon the formation of a nanostructured composite, these characteristic vibrational modes experience a minor blue shift or hypsochromic shift. This indicates that SnO_2_ changes the structure of Ag_3_PO_4_ and there is strong interaction between both phases (Pant et al., 2021; Saud et al., 2017). Based on the FTIR spectra, it can be deduced that the introduction of SnO_2_ does not significantly alter the fundamental composition of Ag_3_PO_4_. One hypothesis suggests that Sn^+4^ ions attach themselves to the negative end of the P-O bond which results in slight shift of the stretching frequency of P-O bonds in nanocomposite. Similar effect has also been observed in other composites of Ag_3_PO_4_ (Selim et al., 2023).

### 3.7 Photocatalytic degradation of isoproturon

**FIGURE 7:**
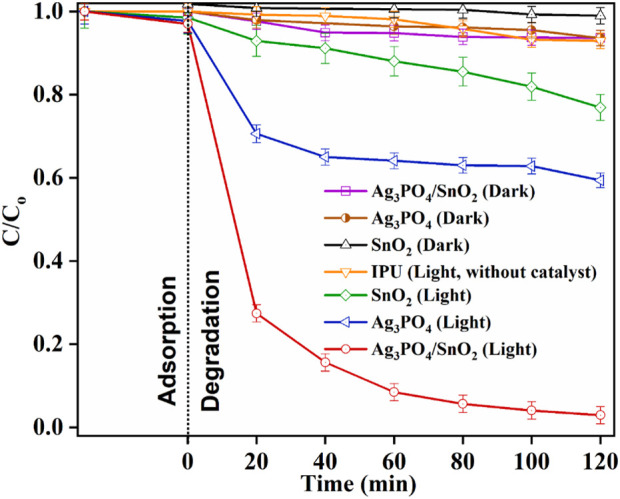
Combined graph showing comparative degradation of IPU.

Ag_3_PO_4_/SnO_2_ NC was used to degrade the IPU solution under UV light irradiation (shown in Figure 7). To attain adsorption-desorption equilibrium, IPU solution is stirred with catalyst in dark for 30 min. Then the solution was irradiated with UV-Visible light for 120 min. Aliquots were collected at 20-min intervals and subjected to centrifugation to remove suspended nanoparticles before UV-visible spectroscopic analysis. Irradiation of IPU solution in absence of photocatalyst resulted in negligible degradation. This owes to the requirement of an efficient photocatalyst. Under UV light irradiation, the degradation efficiency of SnO_2_ NPs was about 25% and that of Ag_3_PO_4_ NPs was 41% with the variation of ± 2%–4%. Compared to them, the composite exhibited the degradation efficiency of 97% with an error bar of ±2%. The higher efficiency of the nanocomposite can be attributed to the larger average surface area and formation of heterojunction that inhibits the electron-hole pair recombination rate. Supplementary Table S2 depicts comparison of synthesized nanocomposite with nano-catalysts reported in literature which have been used for photocatalytic IPU degradation.

**FIGURE 8:**
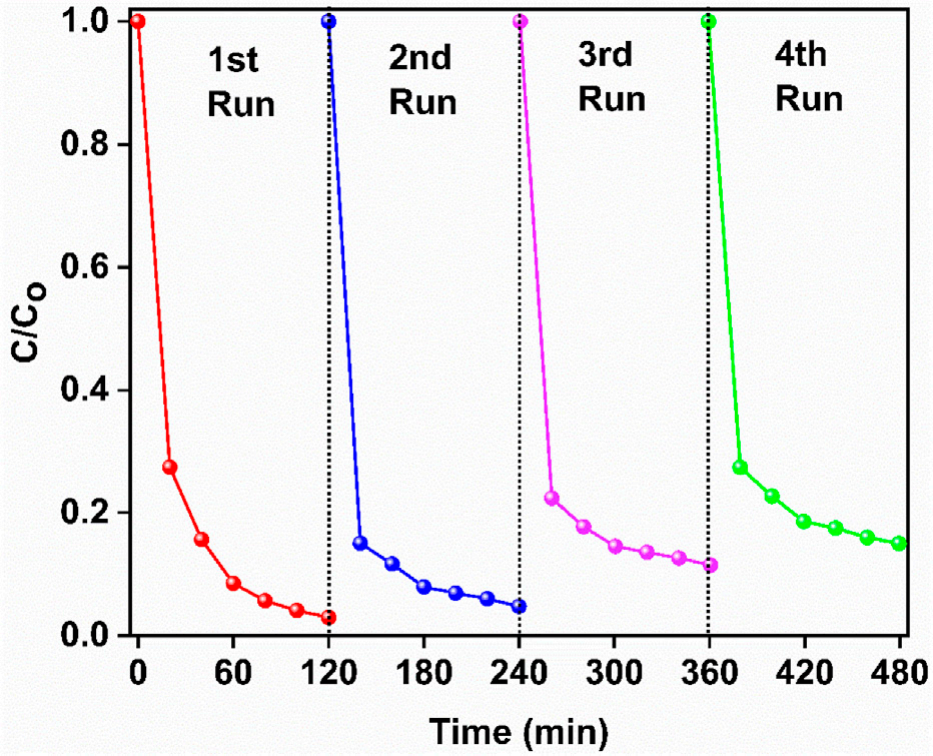
Consecutive photocatalytic degradation of isoproturon using Ag_3_PO_4_/SnO_2_ photocatalyst.

**FIGURE 9:**
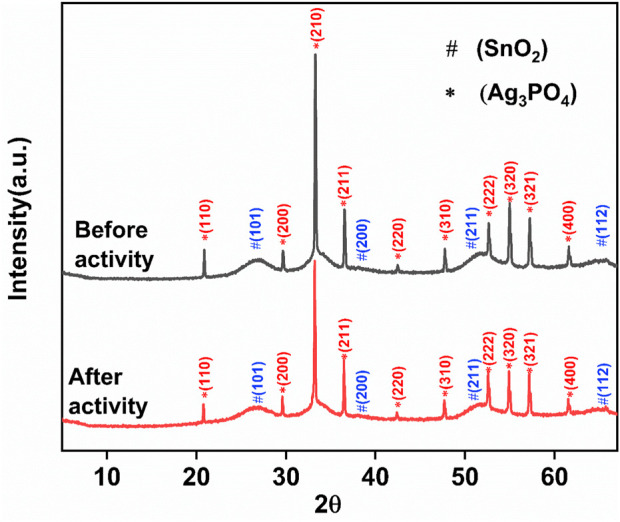
XRD spectrum of the Ag_3_PO_4_/SnO_2_ nanocomposite before and after photocatalytic activity.

SnO_2_/Ag_3_PO_4_ photocatalyst showed high stability and recyclability. In terms of recycling the nanocomposite degraded 87% of IPU even after four consecutive cycles (Figure 8). Recyclability experiments demonstrate the potential of Ag_3_PO_4_/SnO_2_ photocatalyst to be an economical option for IPU degradation. PXRD pattern was taken after four consecutive cycles (Figure 9). It was observed to be unchanged which demonstrate high stability of the nanocomposite.

The degradation mechanism involves the reaction photogenerated holes with water molecules to generate hydroxyl radicals. The conduction band as well as valence band potentials of the SnO_2_ are higher than those of Ag_3_PO_4_ (Niu et al., 2010). As a result, photogenerated electrons in Ag_3_PO_4_ are easily transferred to the surface of SnO_2_, while photoinduced holes stay on the surface of Ag_3_PO_4_, as shown in Figure 13. The band structure of the Ag_3_PO_4_/SnO_2_ photocatalysts must be responsible for the higher photocatalytic activity than pure Ag_3_PO_4_. Furthermore, electronic acceptors such as adsorbed O_2_ may easily capture electrons (which was transferred onto the surface of SnO_2_) to generate a superoxide anion radical $O_{2}^{-•}$ , thus protecting Ag_3_PO_4_ semiconductors from photoreduction (Ag^+^ to Ag). On the contrary, some of these photoinduced holes at the external surface of Ag_3_PO_4_ can be trapped by OH^**−**^ to generate more OH^•^ species (Simonsen, 2014). The formed active species like holes, OH^•^, and $O_{2}^{-•}$ , are responsible for degradation of IPU into less toxic molecules.

Based on the foregoing, it is possible to infer that the suggested Ag_3_PO_4_/SnO_2_ fabrication is an effective and universal technique for developing highly stable and active photocatalysts under UV irradiation.

### 3.8 Trapping experiments

**FIGURE 10:**
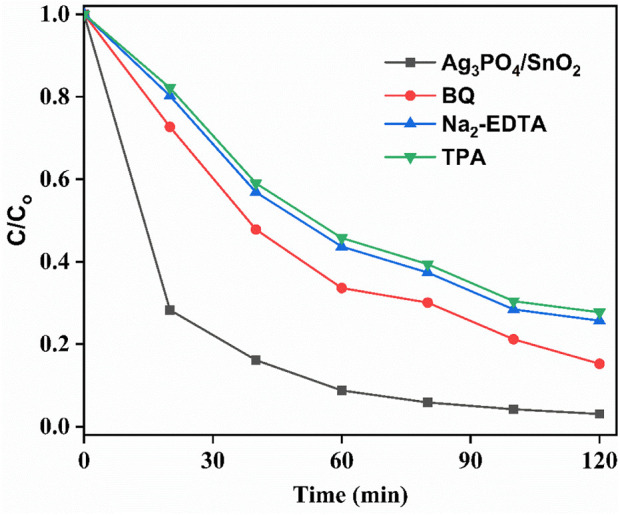
Effect of various trapping/sacrificial agents on degradation efficiency of the photocatalyst.

**FIGURE 11:**
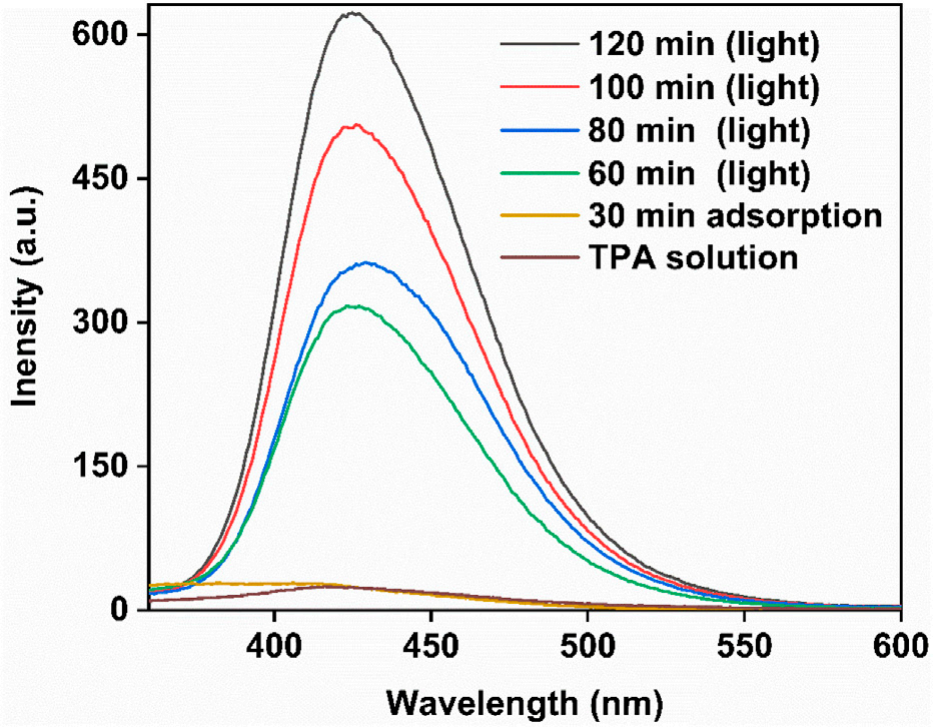
Photoluminescence spectrum of terepthalic acid under light irradiation in presence of Ag_3_PO_4_/SnO_2_.

To explore the species responsible for degradation of IPU, trapping reagents were used. For this purpose, EDTA-Na_2_ (disodium-EDTA), terephthalic acid (TPA) and benzoquinone (BQ) were used for trapping h^+^, OH^•^, and $O_{2}^{-•}$ , respectively. For this purpose, 10 mM solution of each trapping agent was added before starting degradation experiment with IPU solution. After trapping experiments, it was observed that BQ, EDTA-Na_2_ and TPA lowers the degradation efficiency of the catalyst and rendered it to 84.7%, 74.4% and 72.1%, respectively under same experimental conditions during 120 min of light irradiation (Figure 10). This implies that all the *h* ^+^, OH^•^, and $O_{2}^{-•}$ , participate during degradation of IPU with the prepared catalyst but *h* ^+^ and OH^•^, plays vital role during degradation mechanism. To check the formation of OH^•^ during the photocatalytic activity, TPA were used as probe molecule that form a new compound 2-hydroxyterephthalic acid on reacting with OH^•^ which is observed as an absorbance peak at 423 nm in photoluminescence (PL) spectrum (Figure 11). When TPA solution with photocatalyst were stirred under dark for 30 min, no peak was observed at 423 nm in PL spectrum. But under light irradiation, an enhanced peak at 423 nm was observed which became intense with increased irradiation time indicating that OH^•^ concentration increases abundantly throughout the reaction. Also, it is clear from literature that the oxygen involved in photocatalytic degradation of organic water pollutants is dissolved molecular oxygen in water (Li et al., 2011).

### 3.9 Kinetic study

Equations used for kinetic study have been provided in Supplementary Material. Langmuir Hinshelwood and pseudo first order kinetic model are two of the most common models used for photocatalytic degradation processes. Degradation of IPU was followed Langmuir Hinshelwood kinetics and Equation 1 is known as L-H model (Kumar et al., 2008). On integrating Equation 1, we get Equation 2 which represents another form of L-H model in which graph of $\frac{-t}{C_{P}-C_{P,0}}vs\frac{\ln\;\left(\frac{C_{P}}{C_{P,0}}\right)}{C_{P}-C_{P,0}}\;gives\;\frac{1}{k_{\deg}*K}\;as\;a\;slope\;and\;\frac{1}{k_{\deg}}$ as intercept (Tekin et al., 2019).

**FIGURE 12:**
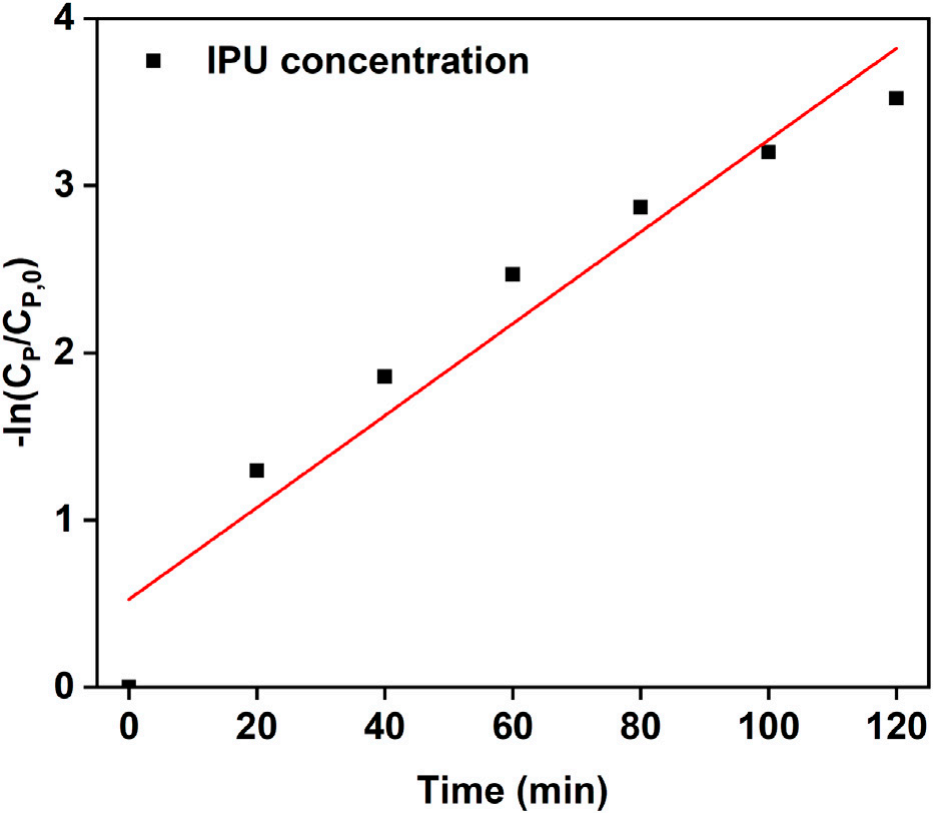
Langmuir–Hinshelwood Kinetics study of IPU degradation.

On the other hand, Equation 3 represents pseudo first order reaction. From literature it has been found that when substrate concentration is lower than 10^–3^ mol L^-1^, KC_P_ << 1 and k_ap_ ∼ k_deg_ K = k_1_ indicating that at very low substrate concentration pseudo first order kinetics is followed (Tekin et al., 2019). In current work it was found that the nanocomposite follows Equation 3 with k_ap_ value of 0.02746 min^-1^. So the degradation rate follows pseudo first order kinetics (Figure 12).

## 4 Identification of intermediates and possible degradation pathways

**FIGURE 13:**
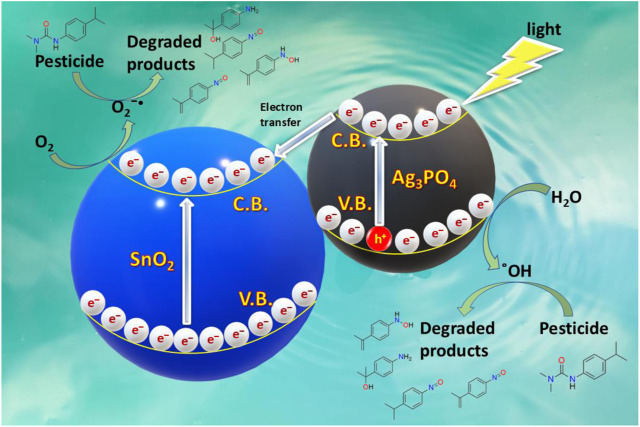
Schematic model for electron transfer in SnO_2_/Ag_3_PO_4_.

The degradation mechanism involves the reaction of photogenerated holes with water molecules to generate hydroxyl radicals. The conduction band as well as valence band potentials of the SnO_2_ are higher than those of Ag_3_PO_4_ (Niu et al., 2010). As a result, photo-excited electrons in Ag_3_PO_4_ are easily transferred to the surface of SnO_2_, while photo-induced holes stay on the surface of Ag_3_PO_4_, as shown in Figure 13. The band structure of the Ag_3_PO_4_/SnO_2_ photocatalysts must be responsible for the higher photocatalytic activity than pure Ag_3_PO_4_. Furthermore, electronic acceptors such as adsorbed molecular O_2_ may easily capture electrons (which was transferred onto the surface of SnO_2_) to generate a superoxide anion radical $O_{2}^{-•}$ , thus protecting Ag_3_PO_4_ semiconductors from photoreduction (Ag^+^ to Ag). On the contrary, some of these photoinduced holes at the external surface of Ag_3_PO_4_ can be trapped by OH^−^ to generate more OH^•^ species (Simonsen, 2014). The formed active species like holes, OH^•^, and $O_{2}^{-•}$ , are responsible for degradation of IPU into less toxic molecules.

**FIGURE 14:**
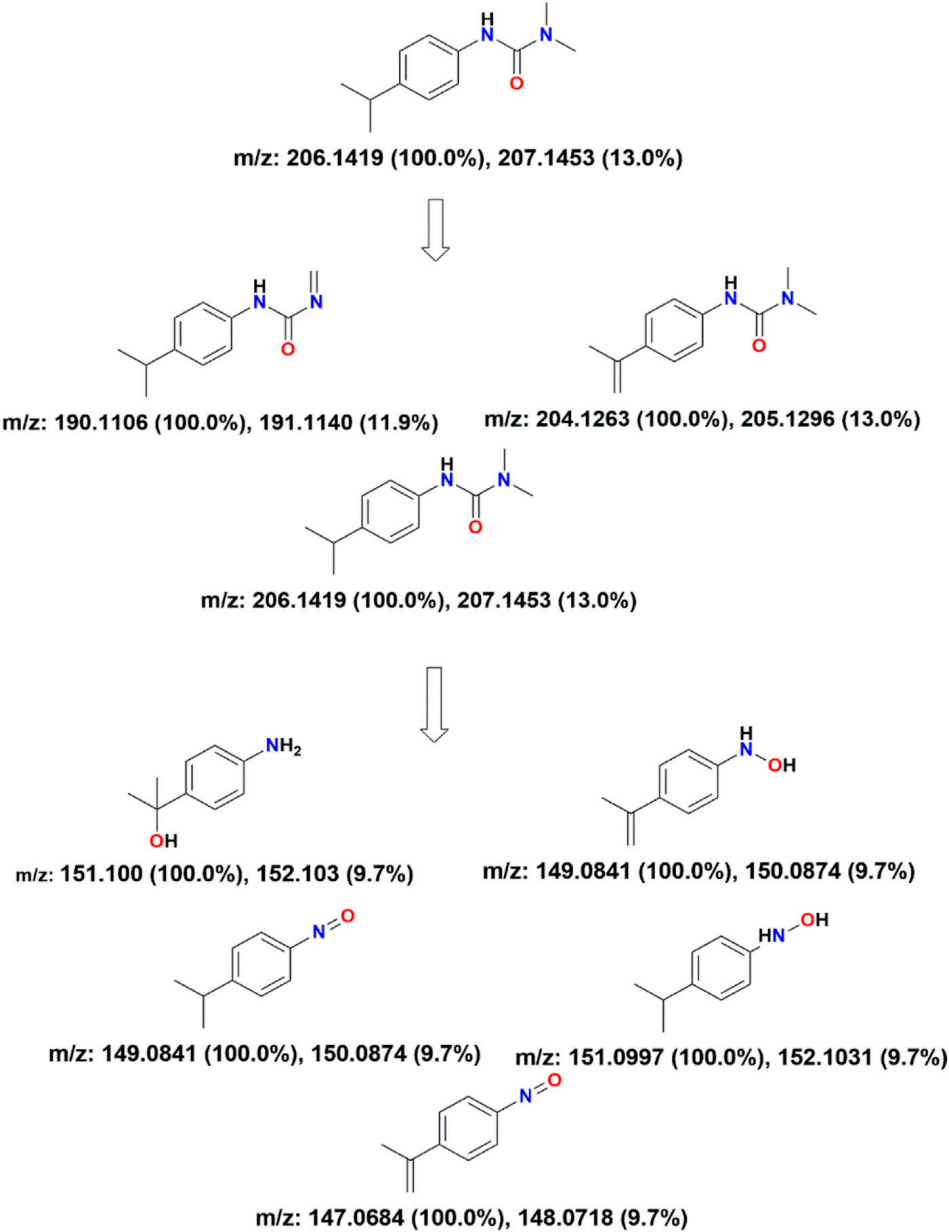
The proposed removal pathways of photodegradation for Isoproturon.

To investigate the degradation pathway of IPU, HRMS was performed to analyse the degradation products and intermediates. Supplementary Figures S4A, B shows the HRMS spectrum of sample before and after UV light irradiation for 120 min, respectively. Based on HRMS analysis, the possible products and the proposed degradation pathway have been shown in Figure 14. The results indicate that the degradation pathway of IPU include reactions such as hydroxylation, demethylation, deamination, oxidation, reduction and decarboxylation (Galichet et al., 2002; Khan et al., 2023; Sharma et al., 2008; Boucheloukh et al., 2017). Photogenerated h^+^, OH^•^, were found to be the main active species responsible for degradation of IPU. The photogenerated e‾ further produced $O_{2}^{-•}$ , and OH^•^ that degrade IPU.

## 5 Conclusion

This work elucidates the successful synthesis, comprehensive characterization, and remarkable photocatalytic degradation of Isoproturon using Ag_3_PO_4_/SnO_2_ nanocomposite. The nanocomposite synthesis was validated using a variety of analytical techniques such as X-ray diffraction, FTIR, SEM, Elemental mapping, DRS and X-ray photoelectron spectroscopy. PXRD spectrum as well as XPS showed successful synthesis of the nanocomposite. DRS showed a decrease in overall band gap of the nanocomposite. Elemental mapping indicates a homogeneous distribution of Ag, P, Sn, and O elements, which further supports the structural integrity of the nanocomposite. The Ag_3_PO_4_/SnO_2_ nanocomposite showed better photocatalytic activity as compared to both pure Ag_3_PO_4_ & pure SnO_2_. The nanocomposite performed approximately 2.3 times greater than its precursor nanoparticles. Formation of heterojunction enhance the light harvesting ability of the nanocomposite and also enhance the charge separation of generated electron-hole pair. This result along with large average surface area of NC responsible in enhanced photocatalytic activity. The mechanistic insights highlight the promise of Ag_3_PO_4_/SnO_2_ nanocomposites for expanded environmental applications in pesticide clean-up, while also providing a deeper understanding of the enhanced photocatalytic activity. Thus, this study opens the door for the creation of effective and long-lasting remedies to the problem of pesticide pollution in the environment.

## Data Availability

The original contributions presented in the study are included in the article/Supplementary Material, further inquiries can be directed to the corresponding authors.
