# Supplementary material for: Synergistic heterojunction effects in Ag3PO4/SnO2 nanocomposites: a photocatalytic study on isoproturon degradation
Source: Front Bioeng Biotechnol. 2025 Apr 4;13:1458965. doi: 10.3389/fbioe.2025.1458965 (PMC12006140; doi:10.3389/fbioe.2025.1458965)
Supplement: Supplementary file 1 [file DataSheet1.pdf]

Synergistic Heterojunction Effects in Ag_3PO_4/SnO_2 Nanocomposites: A Photocatalytic Study on Isoproturon Degradation

Rishi Ram[1], Bhawna[2], Sanjeev Kumar[3], Akanksha Gupta[4], Ravinder Kumar[5*], Kashyap Kumar Dubey[6], Vinod Kumar[3,7*]

[1]School of Physical Sciences, Jawaharlal Nehru University, New Delhi-110067, India; [2]Department of Chemistry, SRM Institute of Science and Technology, Delhi-NCR Campus, Ghaziabad 201204, India; [3]Department of Chemistry, University of Delhi, Delhi-110007, India; [4]Department of Science and Technology, Technology Bhavan, New Delhi-110016, India; [5]Department of Chemistry, Gurukula Kangri (Deemed to be University), Haridwar, Uttarakhand, India; [6]School of Biotechnology, Jawaharlal Nehru University, Delhi, India; [7]Sustainable Energy & Environmental Nanotechnology Group, Special Centre for Nano Science, Jawaharlal Nehru University, Delhi-110067, India.

*Corresponding author: vkumar2@chemistry.du.ac.in; vinod7674@gmail.com; ravinder.kumar@gkv.ac.in

S.I. 1: Synthesis of pure SnO_2 nanoparticles.

S.I. 2: Synthesis of pure Ag_3PO_4 nanoparticles.

S.I. 3: Characterization details.

S.I. 4: Equations used for kinetic studies.

Tables

Table S1: FTIR bands of Ag_3PO_4/SnO_2 nanocomposite.

Table S2: A comparison of synthesized nanocomposite with nano-catalysts reported in literature which have been used for photocatalytic IPU degradation.

Figures

Figure S1: Powder XRD patterns comparison of Ag_3PO_4/SnO_2 nanocomposite, pure Ag_3PO_4 nanoparticles and pure SnO_2 nanoparticles.

Figure S2: W-H plot of Ag_3PO_4/SnO_2 nanocomposite.

Figure S3: Core level XPS spectrum of Phosphorus.

Figure S4: HRMS of isoproturon (a) 0 min and (b) 120 min of light irradiation.

S.I. 1. Synthesis of SnO_2:

The SnO_2 nanoparticles were synthesized using hydrothermal method. 0.11 mmol of $SnCl_2$•$2H_2O$ was prepared in 40 mL of MeOH. Then about 20 mL of H_2O_2 was added slowly in the above solution. Then solution was stirred for 30 minutes. Then the solution was and heated in Teflon-lined stainless-steel autoclave at 150 ^oC for 15 hours. The synthesised nanoparticles were centrifuged, washed with excess methanol and dried in oven at 80 ^oC for 6 hours.

S.I. 2. Synthesis of Ag_3PO_4:

The Ag_3PO_4 nanoparticles were synthesized using hydrothermal method. 2.94 mmol of $AgNO_3$ were dissolved in 30 mL of distilled water. In another beaker 1 mmol of Na_2HPO_4 was dissolved in distilled water. After both the solutions were clear, Na_2HPO_4 solution was added slowly in $AgNO_3$ solution. Formation of yellow coloured precipitate was observed. Then the solution was put in Teflon-lined stainless-steel autoclave and heated at 150 ^oC for 15 hours. The yellow-coloured nanoparticles were centrifuged, washed with distilled water and dried in oven at 80 ^oC for 6 hours.

S.I. 3. Characterization details

For SEM images, Gemini SEM 500 scanning electron microscope (SEM) was used. A Jeol IT200 was used to record the element mapping. Thermo Scientific Nicolet iS50 FTIR tri-detector was used to observe FTIR. Rigaku MiniFlex instrument was used to generate X-ray diffraction (XRD) patterns having Cu filament of K_α =1.54Å and scanning rate of 3 degree per minute at room temperature. BET adsorption-desorption isotherms were performed using Microtrac BELSORP Mix II at 77 K. UV−vis spectra were recorded by Michelson Interferometer FTIR Spectrophotometer. Shimadzu UV-1800 spectrophotometer which utilize a precision Czerny-Turner optical system was used for taking UV−visible diffuse reflectance spectrum. X-ray photoelectron spectroscopy (XPS) was performed by a Shimadzu AXIS Supra$^+$ having Al K_α X-ray.

S.I. 4. Equations used for kinetic studies

$$-\frac{dC_P}{d_t}=\frac{k_{deg}KC_P}{1+KC_P} \tag{1}$$

$$\frac{-t}{C_P-C_{P,0}}=\frac{1}{k_{deg}*K}\times\frac{\ln\left(\frac{C_P}{C_{P,0}}\right)}{C_P-C_{P,0}}+\frac{1}{k_{deg}} \tag{2}$$

$$ln\frac{C_P}{C_{P,0}}=-k_1t \tag{3}$$

Tables:

Table S1: FTIR bands of Ag_3PO_4/SnO_2 nanocomposite

Peak (cm^{-1})	About	Reference
1119	Antisymmetric streching of P-O bonds	(1)
949	PO_4^{3-} stretching	(2)
663	P-O-P Streching	(3)
627	O-Sn-O Stretching	(4)
544	Asymmetric Bending of O=P-O bond	(2)
500	Sn-O Streching	(4)

Table S2: A comparison of synthesized nanocomposite with nano-catalysts reported in literature which have been used for photocatalytic IPU degradation

Catalyst	Synthesis method	Concentration of IPU	Amount of Catalyst	UV lamp power	Degradation efficiency	Time	Ref.
TiO_2 functionalized silica nanofibrous membranes	Electrospinning and dip-coating method	0.5-1 mg/L	-----	300 Watt	100%	8 hours	(5)
SnS_2/RGO nanocomposite	Hydrothermal method	10mL solution of concentration 1ppm	2mg	65 Watt	Rate constant 0.0219 min^{-1}	-----	(6)
GO-TiO_2 catalyst	Hydrothermal method	5mg/L	200mg/L	15 Watt	About 100%	300 min	(7)
PAN/Ag-AgBr@$Bi_{20}TiO_{32}$	Coaxial electrospinning method	15mg/L(50mL solution)	0.1g	500 Watt	87.9%	60 min	(8)
TiO_2	Dip coating method	10mg/L	-----	Two 60 Watt lamps and four 15 watt lamps	57.07%	120 min	(9)

TiO_2/HY composite	Solid state dispersion method	1.14×10^{-4} M(50mL)	50mg	intensity ~75mWcm^{-2}	100%	120min	(10)
Fe-BTC MOF @ aramid fabric (Fe-BTC@AF) composite	layer-by-layer in situ self-assembly methods	5mg/L(50mL)	3cm×3cm	300 watt	90%	7 hours	(11)
Yb^{3+} doped microspherical BiOI	Hydrothermal method	15mg/L(50mL)	50mg	500 watt	90.2%	-----	(12)
Bismuth modified porous silica (Bi_2SiO_5)	Impregnating method	1.14×10^{-4} mol/L		250kW	100%	120 min	(13)
Ag_3PO_4/SnO_2	Hydrothermal method	1ppm	0.1g	125 Watt	97%	120 min	Current work

Figures:

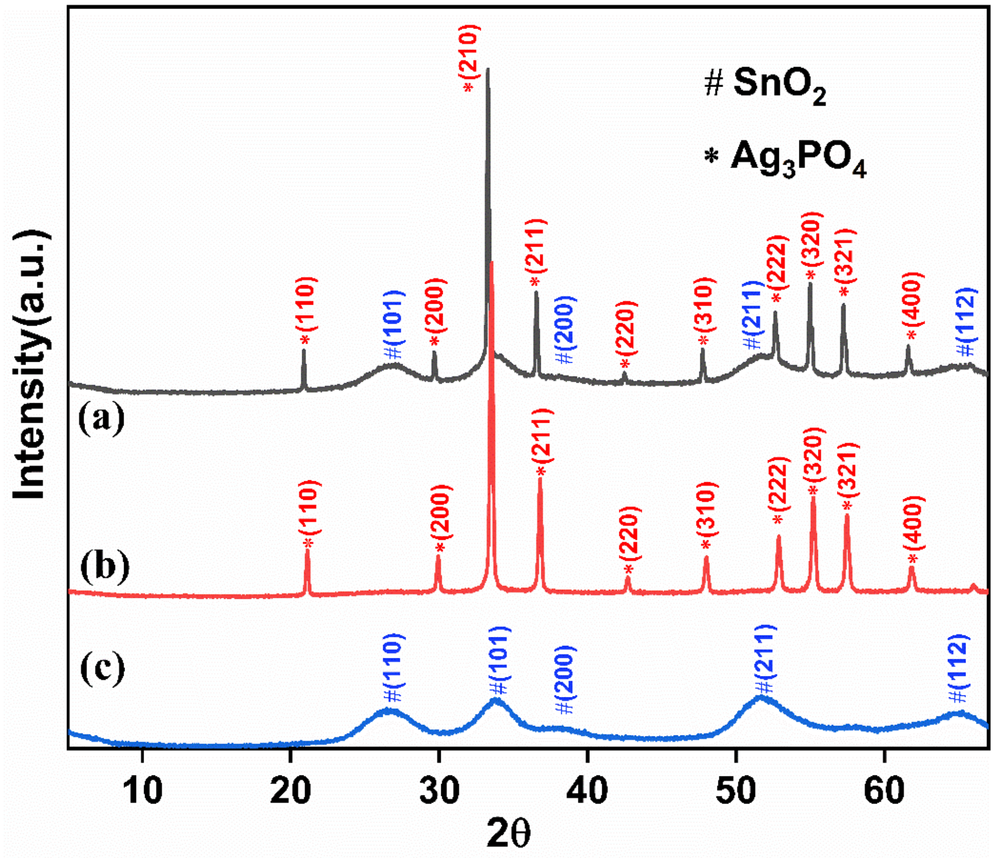

Figure S1: Powder XRD patterns comparison of Ag_3PO_4/SnO_2 nanocomposite, pure Ag_3PO_4 nanoparticles and pure SnO_2 nanoparticles.

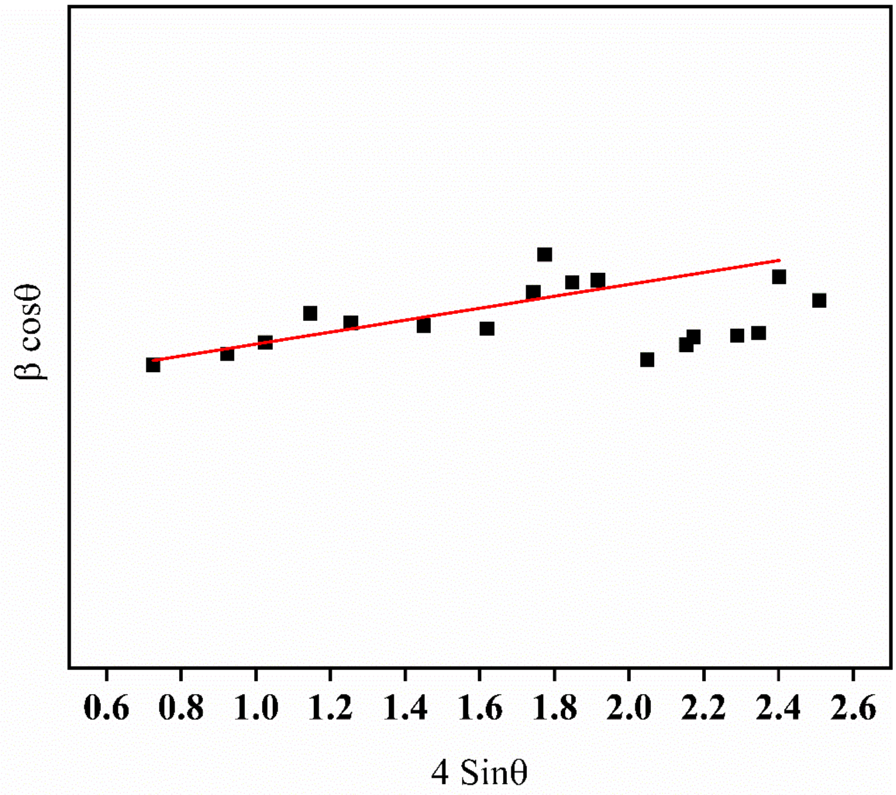

Figure S2: W-H plot of Ag_3PO_4/SnO_2 nanocomposite.

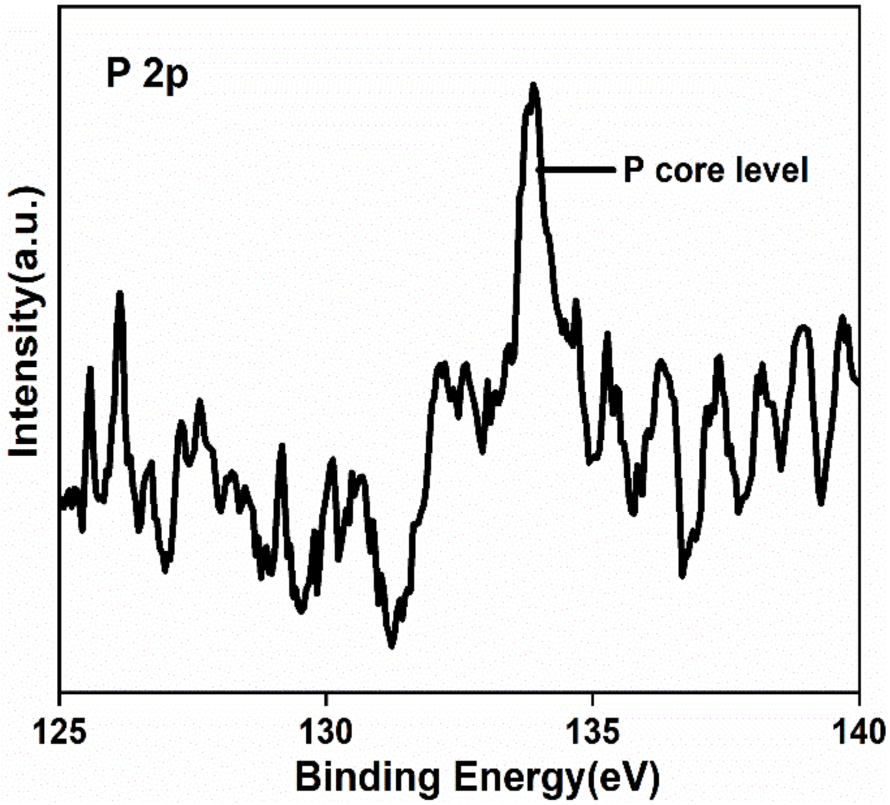

Figure S3: Core level XPS spectrum of Phosphorus.

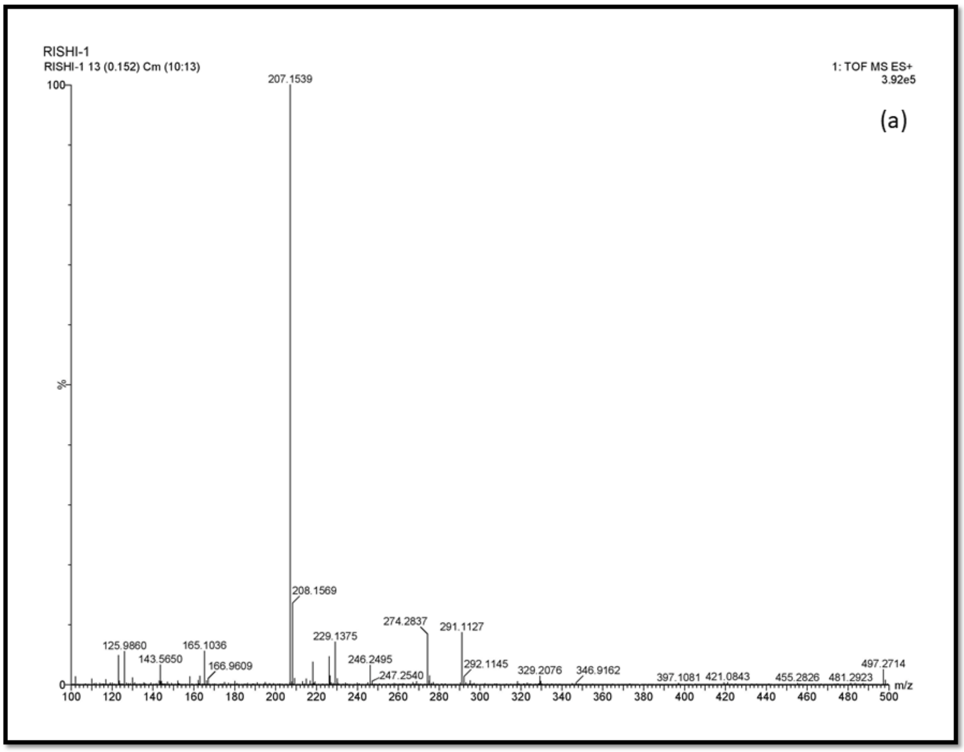

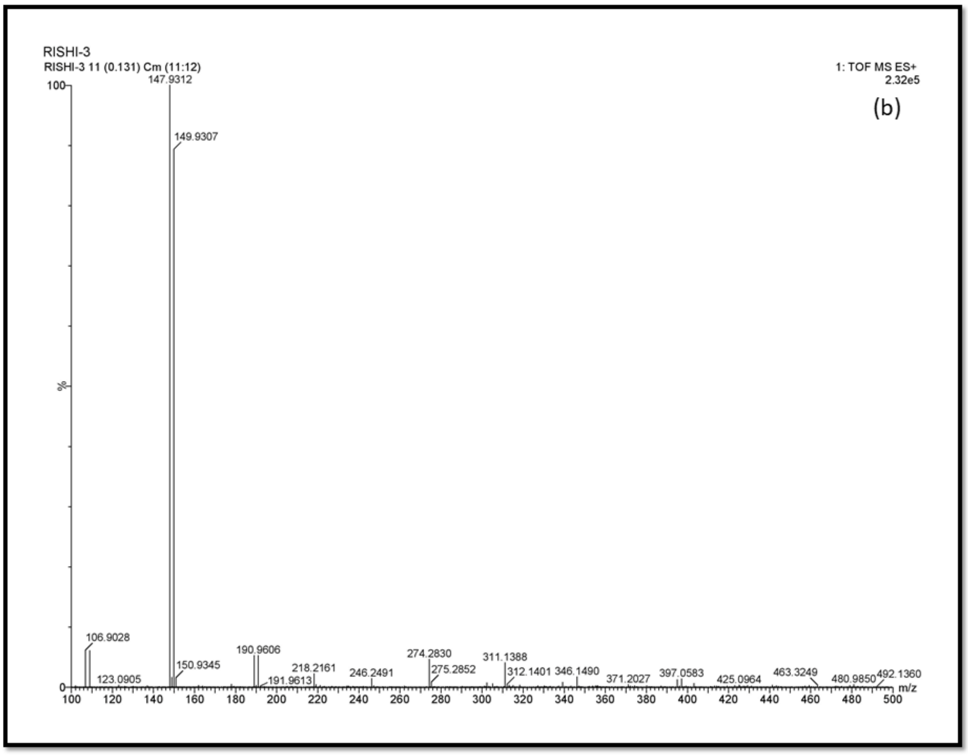

Figure S4: HRMS of isoproturon (a) 0 min and (b) 120 min of light irradiation.

References

1. Kumar S, Surendar T, Baruah A, Shanker V. Synthesis of a novel and stable g-C_3N_4-Ag_3PO_4 hybrid nanocomposite photocatalyst and study of the photocatalytic activity under visible light irradiation. J Mater Chem A. 2013;1(17):5333–40.

2. Nagajyothi PC, Sreekanth TVM, Ramaraghavulu R, Devarayapalli KC, Yoo K, Vattikuti SVP, et al. Photocatalytic dye degradation and hydrogen production activity of Ag_3PO_4/g-C_3N_4 nanocatalyst. J Mater Sci Mater Electron. 2019;30(16):14890–901.

3. Mroczkowska M, Nowinski JL, Zukowska GZ, Mroczkowska A, Garbarczyk JE, Wasiucionek M, et al. Micro Raman, FT-IR/PAS, XRD and SEM studies on glassy and partly crystalline silver phosphate ionic conductors. J Power Sources. 2007;173(2 SPEC. ISS.):729–33.

4. Kumar V, Govind A, Nagarajan R. Optical and photocatalytic properties of heavily F—doped SnO_2 nanocrystals by a novel single-source precursor approach. Inorg Chem. 2011;50(12):5637–45.

5. Loccufier E, Deventer K, Manhaeghe D, Van Hulle SWH, D'hooge DR, De Buysser K, et al. Degradation kinetics of isoproturon and its subsequent products in contact with TiO_2 functionalized silica nanofibers. Chem Eng J. 2020;387(September 2019).

6. Dashairya L, Sharma M, Basu S, Saha P. SnS_2/RGO based nanocomposite for efficient photocatalytic degradation of toxic industrial dyes under visible-light irradiation. J Alloys Compd. 2019;774:625–36.

7. Luna-Sanguino G, Ruíz-Delgado A, Tolosana-Moranchel A, Pascual L, Malato S, Bahamonde A, et al. Solar photocatalytic degradation of pesticides over TiO_2-rGO nanocomposites at pilot plant scale. Sci Total Environ. 2020;737:140286.

8. Mao Z, Xie R, Fu D, Zhang L, Xu H, Zhong Y, et al. PAN supported Ag-AgBr@$Bi^{20}TiO_{32}$ electrospun fiber mats with efficient visible light photocatalytic activity and antibacterial capability. Sep Purif Technol. 2017;176:277–86.

9. Espino-Estévez MR, Fernández-Rodríguez C, González-Díaz OM, Navío JA, Fernández-Hevia D, Doña-Rodríguez JM. Enhancement of stability and photoactivity of TiO_2 coatings on annular glass reactors to remove emerging pollutants from waters. Chem Eng J. 2015;279:488–97.

10. Sharma MVP, Lalitha K, Durgakumari V, Subrahmanyam M. Solar photocatalytic mineralization of isoproturon over TiO_2/HY composite systems. Sol Energy Mater Sol Cells. 2008;92(3):332–42.

11. Zhang H, Wu S, Zhang Y, Mao Z, Zhong Y, Sui X, et al. Fabrication of Fe-BTC on aramid fabrics for repeated degradation of isoproturon. Environ Sci Pollut Res. 2023;30(12):35214–22.

12. Zhang L, Ma Z, Xu H, Xie R, Zhong Y, Sui X, et al. Preparation of upconversion Yb^{3+} doped microspherical BiOI with promoted photocatalytic performance. Solid State Sci. 2018;75:45–52.

13. Police AKR, Basavaraju S, Valluri DK, Machiraju S. Bismuth modified porous silica preparation, characterization and photocatalytic activity evaluation for degradation of isoproturon. J Mater Sci Technol. 2013;29(7):639–46.
